# Comparison of the efficacy of two natural surfactants (BERAKSURF and BLES) in the treatment of respiratory distress syndrome among preterm neonates

**DOI:** 10.1186/s12887-023-04406-2

**Published:** 2023-12-01

**Authors:** Fatemeh Sabzevari, Mahdie Eslamian, Fatemeh Karami Robati, Bahareh Bahmanbijari, Zahra Daei Parizi, Zahra Jamali

**Affiliations:** 1 Department of Pediatrics, School of Medicine, Afzalipour Hospital, Kerman University of Medical Sciences, Kerman, Iran; 2 Clinical Research Development Unit, Afzalipour Hospital, Kerman University of Medical Sciences, Kerman, Iran

**Keywords:** Beraksurf®, Premature birth, Respiratory distress syndrome, Pulmonary surfactants, Therapy

## Abstract

**Background:**

The benefit of surfactant replacement therapy for respiratory distress syndrome (RDS) has been demonstrated. However, some surfactants are expensive and usually inaccessible. Consequently, the Iranian Survanta was produced, but its effect on complications and mortality of RDS is unknown. This study aimed to compare the therapeutic outcomes of Iranian surfactant (beraksurf) and BLES (bovine lipid extract surfactant) on RDS treatment among preterm neonates.

**Methods:**

This triple blinded randomized controlled trial study was performed on 128 eligible neonates diagnosed with RDS in Afzalipour hospital in Kerman, Iran. Diagnosis of RDS, gestational age of 28–34 weeks and weight ≥ 1 kg were considered as inclusion criteria. Congenital anomalies such as congenital cyanotic heart diseases, digestive system anomalies and chromosome abnormalities were the exclusion criteria Neonates were randomly assigned into two equal groups: (1) those treated with BLES (n = 64) and (2) those treated with beraksurf (n = 64). Complications including patent ductus arteriosus (PDA), sepsis, intraventricular hemorrhage (IVH), pneumothorax, pulmonary hemorrhage, mortality, and also, the number of days required for invasive mechanical ventilation (using ventilator) and non-invasive continuous positive airway pressure (CPAP) were evaluated for all neonates. The risk ratio (RR) was calculated at 95% of confidence intervals (CI).

**Results:**

Compared with BLES group, the RR estimate among neonates in beraksurf group was 0.89 (0.66–1.20) for PDA, 0.71 (0.23–2.13) for IVH, 0.44 (0.14–1.36) for sepsis, 0.35 (0.13–0.93) for pneumothorax, 0.33 (0.12–0.86) for pulmonary hemorrhage, and 0.55 (0.28–1.05) for mortality.

**Conclusions:**

Despite advances in the use of exogenous surfactants for the treatment of neonatal respiratory distress syndrome; There are still some controversial topics in this field. The results obtained in the present study showed that the two types of surfactant (BERAKSURF and BLES) have similar efficacy for the treatment and short-term outcomes in preterm infants with respiratory distress syndrome. Therefore, due to the cost-effectiveness of BRAKSURF compared to BLES, We recommend choosing BERAKSURF in terms of treatment.

**Supplementary Information:**

The online version contains supplementary material available at 10.1186/s12887-023-04406-2.

## Background

Respiratory distress syndrome (RDS) is a prevalent cause of morbidity and mortality in premature neonates [1] usually within the first hours after birth [2]. Among the important risk factors for RDS are low gestational age and low birth weight [3]. Septicemia, bronchopulmonary dysplasia, patent ductus arteriosus (PDA), pulmonary hemorrhage, apnea, blood pressure disorders, growth failure, intraventricular hemorrhage (IVH), and leukomalacia are the most prevalent complications of RDS [4], and are caused by surfactant reduction. Hypoxia follows from atelectasis, loss of functional residual capacity, and ventilation perfusion mismatch (V/Q defect), which occur as a result of surfactant reduction [2].

Normally, surfactant is present in the amniotic fluid from the 28th week of gestation, and its production depends on normal pH, temperature, and perfusion [5]. Some conditions such as asphyxia, hypoxia, and ischemia especially with hypovolemia, hypotension, and cold stress lead to suppression of surfactant production [6]. Given these complications, studies concluded that surfactant replacement therapy is a necessity.

So far, there are many studies that have evaluated the effect of natural and synthetic surfactants on RDS treatment [7]. The surfactant replacement therapy reduced neonate mortality and led to improved symptoms, although some complications such as hypoxia, bradycardia, and bronchopulmonary risk were present. So, the use of various types of surfactants including natural ones such as, Infasurf, Curosurf, and Survanta and synthetic ones such as Surfaxin and Exosurf was amended [8]. These surfactants are expensive, and are not available for everyone especially in countries like Iran. Based on this issue, an effective surfactant with lower cost and more availability is necessary. The first Iranian surfactant called Beraksurf was made with Survanta pharmaceutically active substance. However, the effect of this surfactant on treatment of RDS and its complications is not exactly clear.

A clinical trial in the United States comparing the effect of survanta surfactant and curosurf in premature babies with an average gestational age of 26–29 weeks and an average weight of 1400 g, reported a decrease in supplemental oxygen in babies receiving curosurf as well as the prevalence of patent ductus arteriosus in babies receiving curosurf Compared to surventa, it was reported less with a statistically significant difference [9].

Another clinical trial was conducted in Zika-free NICU of Turkey in infants less than 37 weeks gestational age from July 2008 to June 2009. 126 premature babies with RDS in two groups had similar basic demographic characteristics; In this study, the group receiving survanta surfactant required two or more doses compared to treatment with Corosurf; Also, extubation in the corosurf group was more within the first three days of drug administration, and the need for Fio2 after treatment on the first, third and fifth days was significantly lower in the corosurf group. Although the rate of mortality and morbidity of the two groups were similar, the findings of the study indicated an increase in the chance of survival in the corosurf group [10].

This clinical trial study is designed to compare the effect of the Canadian BLES with the Iranian Survanta as an efficient surfactant.

## Methods

### Study population

This triple-blind randomized clinical trial study was performed on hospitalized neonates in neonatal intensive care unit (NICU) of Afzalipour hospital (Kerman, Iran) between April 1, 2020 and February 1, 2021. Diagnosis of RDS, gestational age of 28–34 weeks and weight ≥ 1 kg were considered as inclusion criteria. Congenital anomalies such as congenital cyanotic heart diseases, digestive system anomalies and chromosome abnormalities were the exclusion criteria.

RDS diagnosis and its severity was conducted based on classic clinical presentation of RDS includes grunting respirations, retractions, nasal flaring, cyanosis, and increased oxygen requirement, together with diagnostic finding and onset of symptoms shortly after birth [11].

### Study design

**Fig. 1:**
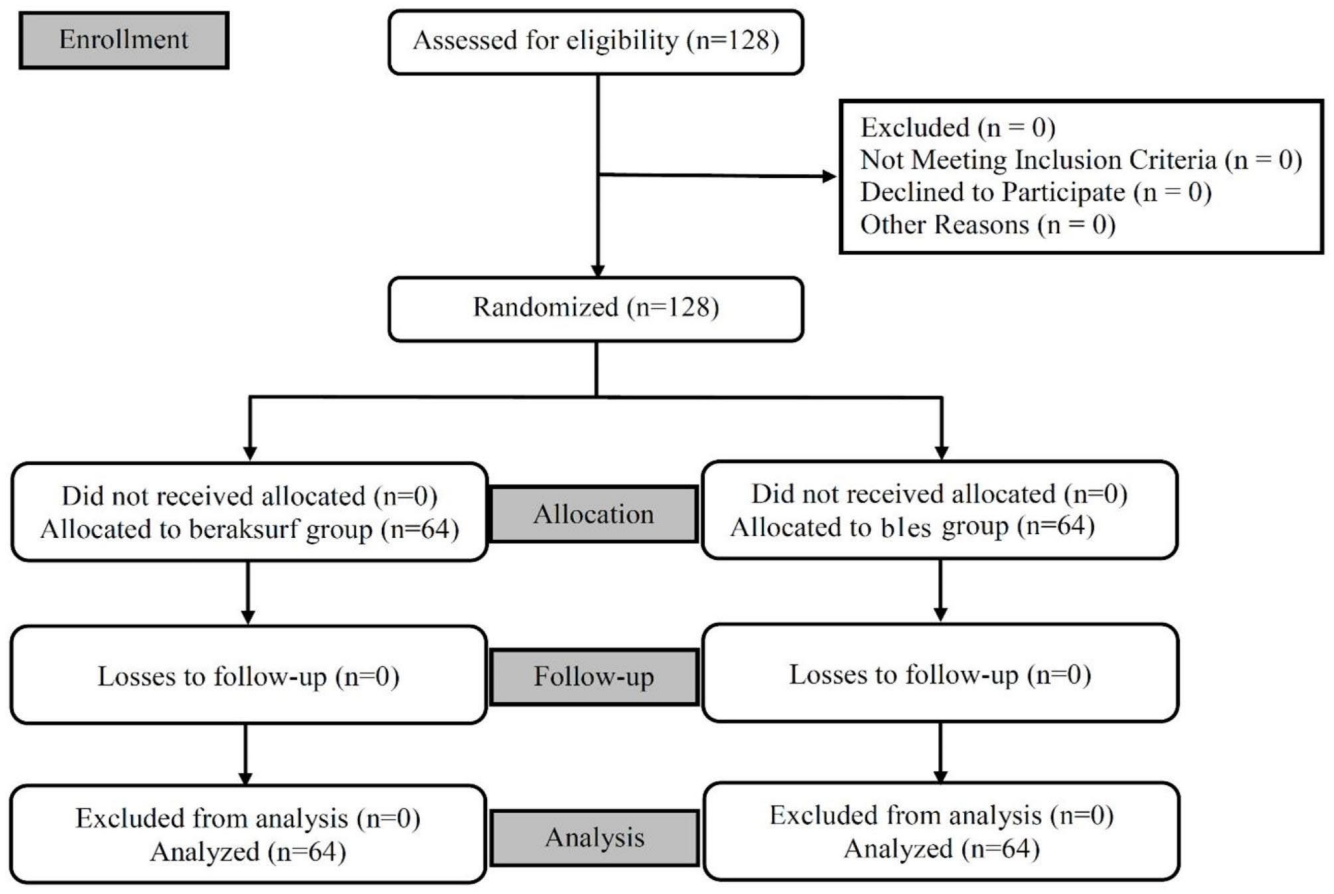
Flowchart of patients’ enrollment in the two different groups

Sample size was calculated at 95% confidence interval (CI) and 80% power based on a similar study [12] with standard deviation (SD) 3.7 using Stata software version 12. Sixty-four participants were assigned randomly to each group (Fig. 1).

### Randomization and allocation concealment

A random number sequence was generated by a person who was not involved in the study, using computer random sequence Stata software version 12. Allocation concealment was performed using numbered, opaque, and sealed envelopes that were opened by the primary researcher at the randomization time. Therefore, all participants had equal chances of being assigned to any group.

### Blinding

The neonates, data collectors, assessors, and analysts were unaware of the type of surfactant in each group and were blinded, making this study triple-blind.

### Intervention

Two types of surfactants were used for intervention in this study. One group received BLES 135 mg/kg (produced by Blesbiochemical, Canada) and the other group received Survanta (Beraksurf®) 100 mg/kg (produced by Tekzima, Iran). Drug administration in both groups was performed intratracheally for 10 min. The first dose was given immediately after hospitalization, and if it was not required at that time, it was prescribed in the hospitalization period and with deterioration of RDS (this deterioration was not justifiable with other causes).

Chest radiography was performed in both groups 6 h after the first dose. Based on radiographic findings and neonate clinical condition, second and third dose were either prescribed or unfixed.

### Outcomes

The number of days required for the use of invasive mechanical ventilation (ventilator) and non-invasive continuous positive airway pressure (CPAP) was recorded for all neonates. Some complications were evaluated. These complications are PDA (assessed by echocardiography), IVH (assessed by sonography), sepsis (using blood culture), and pneumothorax (using chest X-ray; and assessed by clinical symptoms) and pulmonary hemorrhage (using chest X-ray; and assessed by clinical symptoms). The mortalities in both groups were also recorded.

### Statistical analysis

Data analysis was performed with Stata software version 12 (StataCorp, College Station, TX, USA), and *P* value < 0.05 was considered as statistically significant. The analysis was performed in two descriptive and inferential sections. Descriptive, quantitative and categorical data were presented as mean ± standard deviation (minimum–maximum) and frequency (percentage), respectively. Based on quantitative data of comparison between the 2 groups, analyses were performed using independent sample t-tests. Additionally, analyses based on categorical data between the 2 groups were done using Chi-square test. Also, the risk ratio (RR) was calculated for complication and mortality of RDS.

## Results

The mean of the gestational age of neonates in Beraksurf and BLES surfactant groups were 31.64 ± 1.69 weeks and 31.03 ± 1.96 weeks and the difference was not significant (*P =* 0.0640). The mean of the collective birth weights of neonates in Beraksurf group was 1664.53 ± 631.27 kg and 1625.55 ± 567.86 kg in the BLES group. There was no significant difference between the mean of birth weight in both groups (*P =* 0.646). Baseline and demographic information of the two groups is demonstrated in Table 1.

**Table 1:** Baseline and Demographic Information

Variables	Type of surfactant			Total (n = 128)
	Beraksurf (n = 64) N (%)	BLES (n = 64) N (%)	*P* value	
**Gender (%)**				
Female	23 (35.9)	26 (40.6)	0.585	49 (38.3)
Male	41 (64.1)	38 (59.4)		79 (61.7)
**Type of delivery**				
Vaginal	14 (21.9)	21 (32.8)	0.165	35 (27.3)
Cesarean	50 (78.1)	43 (67.2)		93 (72.7)
**Diabetes in mother**				
No	51 (79.7)	45 (70.3)	0.221	96 (75.0)
Yes	13 (20.3)	19 (29.7)		32 (25.0)
**Steroid in mother**				
No	8 (12.5)	17 (26.6)	0.045	25 (19.5)
Yes	56 (87.5)	47 (73.4)		103 (80.5)
	Means ± SD	Means ± SD		
**Gestational age (Week)**	31.64 ± 1.69	31.03 ± 1.96	0.064	
**Birth weight (kg)**	1664.53 ± 361.27	1625.55 ± 567.86	0.646	

* P−value was calculated using chi−2 statistic test at 95% level of confidence interval and independent t−test

The mean time to receive surfactant was 4.49 ± 1.18 h in Beraksurf group and 4.24 ± 1.09 in BLES group. Time of receipt of surfactant in Beraksurf group was ≤ 12 h in 35 (54.7%), 2–12 h in 25 (39.1%) and ≥ 12 h in 4 (6.2%) neonates. In BLES group, it was ≤ 12 h in 35 (54.7%), 2–12 h in 27 (42.2%), and ≥ 12 h in 2 (3.1%) neonates. There was no significant difference between the mean post-natal age of surfactant administration in both groups (*P* ***=*** 0.689). In the Beraksurf group, 83.9% (n = 52) of neonates received one dose and 16.1% (n = 10) received two doses of surfactant, while in BLES group, 79.7% (n = 51) of neonates received one dose, 18.8% (n = 12) received two doses, and 1.6% (n = 1) received three doses of surfactant. There was no significant difference in the frequencies of surfactant administration between the two groups (*P =* 0.560).

The mean days of hospital stay were 17.66 ± 1.46 days and 16.68 ± 1.46 days in Beraksurf and BLES groups respectively; however, this difference was not significant (*P* = 0.641). Neonates that received Beraksurf spent a mean of 4.39 ± 0.74 days with ventilation support and those that received BLES ventilated for a mean of 4.46 ± 0.64 days, but the difference was not significant (*P* = 0.937). Also, the mean of days required for a CPAP in Beraksurf group was 4.79 ± 0.48 days which was higher than that of neonates in BLES group at 4.1 ± 0.49 days. This difference was also not significant (*P* = 0.325).

Table 2 shows the comparison of the effects of the two types of surfactants on complications among neonates. The differences between frequencies of pneumothorax (*P* = 0.025) and pulmonary hemorrhage (*P* = 0.015) in the two groups were statistically significant. The incidence risk of all complications in Beraksurf group was lower than BLES group so that the incidence risk of pneumothorax and pulmonary hemorrhage were 0.35 (95% CI: 0.13–0.93) and 0.33 (95% CI: 0.12–0.86) in Beraksurf group compared with BLES group. Other risk differences were not significant.

**Table 2:** Comparison of effects of the two types of surfactants on complications among neonates

Variables	Type of surfactant			RR (95% CI)
	Beraksurf	BLES	*P* value	
**PDA (%)**				
No	29 (45.3)	25 (39.1)	0.474	0.89 (0.66–1.20)
Yes	35 (54.7)	39 (60.9)		
**IVH (%)**				
No	59 (92.2)	57 (89.1)	0.544	0.71 (0.23–2.13)
Yes	5 (7.8)	7 (10.9)		
**Sepsis (%)**				
No	60 (93.8)	55 (85.9)	0.143	0.44 (0.14–1.36)
Yes	4 (6.3)	9 (14.1)		
**Pneumothorax (%)**				
No	59 (92.2)	50 (78.1)	0.025	0.35 (0.13–0.93)
Yes	5 (7.8)	14 (21.9)		
**Pulmonary hemorrhage (%)**				
No	59 (92.2)	49 (76.6)	0.015	0.33 (0.12–0.86)
Yes	5 (7.8)	15 (23.4)		
**Mortality (%)**				
No	53 (82.8)	44 (68.8)	0.063	0.55 (0.28–1.05)
Yes	11 (17.2)	20 (31.3)		

* *P* value was calculated using chi−2 statistics at 95% level of confidence interval

RR: risk ratio, PDA: patent ductus arteriosus, IVH: intraventricular hemorrhage

## Discussion

The prevalence of RDS is 45% [13], and without proper treatment in the first days after labor, RDS deteriorates rapidly and increases mortality in neonates [14]. According to some studies, exposure to oxygen in full term neonates increases their oxidative stress at least for a few months [15]. Thus, prionflammatory cytokines are increased significantly when the alveolar macrophages in a premature neonate’s lungs are exposed to oxygen [16]. These cytokines are increased in preterm neonates with chronic lung diseases. So, mortality in neonates with RDS is high, even as ventilators are used [17]. Surfactant replacement therapy is a standard procedure for RDS treatment in neonates. Surfactants are extracted mainly from lipids of bovine lungs and contain various amounts of lipophilic surface protein [18]. Treatment with surfactant decreases the mortality in neonates and as yet, natural surfactants have better prognoses than synthetic surfactants [19]. According to a meta-analysis study, the natural surfactants decreased pneumothorax and mortality compared to synthetic types [20]. All surfactant replacement therapy regimens lead to improved oxygenation and decreased air leaks in premature neonates under ventilator therapy. Also, treatment with surfactants decreases the total mortality in the ventilated neonates. Despite the different chemical components of various surfactants, all of them are effective in RDS prognosis [2]. Although, finding an effective surfactant with lower costs and side effects is necessary.

In this study, we compared a new and cost-effective surfactant produced in Iran with a relatively more expensive and inaccessible surfactant. Based on our results, there were no significant differences between frequencies of gender, type of delivery and diabetes in mothers in BLES and Beraksurf groups. In the present study, neonates treated with Beraksurf experienced the complications such as PDA, IVH, sepsis, pneumothorax, and pulmonary hemorrhage less frequently than those treated with BLES. From all these assessed complications, only pneumothorax and pulmonary hemorrhage caused significant statistical difference between the two groups. Also, the mortality rate in Beraksurf group was 17.2% and was lower than BLES group (31.3%), however, the *P* value of its difference was borderline. Compared with BLES group, the RR estimates of pneumothorax and pulmonary hemorrhage were 0.35 and 0.33 in Beraksurf group and were significant. Also, the incidence risk of other complications in Beraksurf group was lower than BLES group and were not significant. There was no significant difference between mean of hospital stay, ventilator, and CPAP days in the two groups.

The Beraksurf treatment in our study is a cost-effective and available surfactant; based on our results, its effect on RDS complications is similar to BLES. When compared with BLES, incidence risk of some complications such as pneumothorax and pulmonary hemorrhage in RDS patients is lower with this surfactant. BLES was first used in 1983; this Canadian surfactant is a powerful and effective drug in RDS treatment and is a fully natural human surfactant produced from amniotic fluid or biosynthetic materials. But, the Survanta is a natural corrected surfactant that is produced in some countries. In Iran this surfactant with the brand name Beraksurf® was produced by Tekzima pharmaceutical company. The chemical component of Beraksurf is similar to other Survanta and it is expected to be an effective surfactant with lower price and more availability.

Some studies compared the non-Iranian Survanta with other surfactants (given the similarity of Iranian Survanta to Survanta manufactured in other countries). In a study conducted by Macooie et al. [21], the effects of BLES and Survanta on RDS treatment were compared. The results of this study showed that there was no difference between frequencies of complications and mortalities in the two groups. In a clinical trial study [22], the effectiveness and safety of two types of natural surfactants, Curosurf and Survanta, were assessed. There was no difference between RDS complications and time of CPAP in the two groups. In Mousavi et al.’s study [23], the effects of three natural surfactants Curosurf, Survanta, and Alveofact on RDS treatment in neonates were evaluated. The results showed that there were no differences between clinical parameters such as IVH, hospital stay length, and need for ventilator before and after surfactant therapy. But based on the comparison between Survanta and Alveofact, the incidence risk of pneumothorax and pulmonary hemorrhage in Survanta group was significantly lower than Alevofact group. Their study concluded that Survanta surfactant was more effective for RDS treatment. Hence Mousavi et al.’s study matched our findings regarding lower incidence risk of pneumothorax and pulmonary hemorrhage in neonates treated with Survanta. Also, in a cross-sectional study conducted by Hashemiannejad et al. [24], efficacy of Survanta was higher and its CPAP time was lower than Curosurf and Newfactan surfactant. In a study conducted by Ramanathan et al. [12], the effect of Curosurf and Survanta surfactants was assessed on RDS treatment in preterm neonates. This study concluded that there was no significant difference between the effects of these two surfactants. Similar effects of Beraksurf and BLES surfactants on some RDS complications and also hospital stay length and CPAP time in our study are in line with Ramanathan’s study. In a study conducted by Fallahi et al. [25], the mortality rate in RDS neonates with Survanta group was higher than Curosurf group. However, there was no difference between frequencies of pneumothorax, PDA, pulmonary hemorrhage, IVH, and ventilator time in the two groups. In another study, results similar to our study were found with no significant difference between frequencies of PDA, IVH, sepsis, and mortality in RDS neonates treated with Survanta compared with poractant alfa [26]. But in Malloy et al.’s study, the prevalence of PDA in RDS neonates treated with poractant alfa was lower than Survanta. However, other complications were not different between the two groups [27]. Considering the effectiveness of Iranian surfactant, our results are in line with the findings of the latter two studies. Therefore, the Survanta is an important and effective surfactant for RDS treatment in neonates.

## Conclusions

Considering the effectiveness of neonatal RDS treatment and ventilator-induced complications in these patients, we recommend the use of surfactant replacement therapy. Our scientific study assessed Beraksurf®, an Iranian Survanta, as effective as international surfactant products on neonatal RDS treatment and safe enough to be prescribed. Hence, we suggest the clinicians to consider it as an alternative when international brands are more expensive and inaccessible.

### Electronic supplementary material

Below is the link to the electronic supplementary material.

Supplementary Material 1

Supplementary Material 2

## Data Availability

All data generated or analyzed during this study are included in this published article.

## List of abbreviations

RDS: respiratory distress syndrome
PDA: patent ductus arteriosus
IVH: intraventricular hemorrhage
CPAP: continuous positive airway pressure
RR: risk ratio
CI: confidence intervals
NICU: neonatal intensive care unit
SD: standard deviation
