# Supplementary material for: Comparison of the efficacy of two natural surfactants (BERAKSURF and BLES) in the treatment of respiratory distress syndrome among preterm neonates
Source: BMC Pediatr. 2023 Dec 1;23:608. doi: 10.1186/s12887-023-04406-2 (PMC10691073; doi:10.1186/s12887-023-04406-2)
Supplement: Supplementary file 2 — Supplementary Material 2 [file 12887_2023_4406_MOESM2_ESM.docx]

**Project summary**

The benefit of surfactant replacement therapy for respiratory distress syndrome (RDS) has been demonstrated. However, some surfactants are expensive and usually inaccessible. Consequently, the Iranian Survanta was produced, but its effect on complications and mortality of RDS is unknown. This study aimed to compare the therapeutic outcomes of Iranian Survanta and BLES (bovine lipid extract surfactant) on RDS treatment among preterm neonates.

This triple blinded randomized controlled trial study was performed on 128 eligible neonates diagnosed with RDS in Afzalipour hospital in Kerman, Iran. Neonates were randomly assigned into two equal groups: 1) those treated with BLES (n=64) and 2) those treated with Survanta (n=64). Complications including patent ductus arteriosus (PDA), sepsis, intraventricular hemorrhage (IVH), pneumothorax, pulmonary hemorrhage, mortality, and also, the number of days required for invasive mechanical ventilation (using ventilator) and non-invasive continuous positive airway pressure (CPAP) were evaluated for all neonates. The risk ratio (RR) was calculated at 95% of confidence intervals (CI).

Compared with BLES group, the RR estimate among neonates in Survanta group was 0.89 (0.66-1.20) for PDA, 0.71 (0.23-2.13) for IVH, 0.44 (0.14-1.36) for sepsis, 0.35 (0.13-0.93) for pneumothorax, 0.33 (0.12-0.86) for pulmonary hemorrhage, and 0.55 (0.28-1.05) for mortality.

According to the results of this study, Beraksurf® as an Iranian Survanta is a cost-effective and accessible surfactant for RDS treatment in neonates.

**General information**

- This study was supported by the pediatric health research center at the Kerman University of Medical Sciences.
- Fateme Sabzevari designed the initial concept, prepared the manuscript, and helped with the first draft of the manuscript. Bahareh Bahmanbijari, Zahra Daei parizi and Zahra Jamali designed the initial concept. Mahdie Eslamian participated in manuscript preparation and data collection. Fatemeh Karami Robati participated in revising manuscript. Address: Department of Pediatrics, School of Medicine, Afzalipour Hospital, Kerman University of Medical Sciences, Kerman, Iran.

**Rationale & background information**

The surfactant replacement therapy reduced neonate mortality and led to improved symptoms, although some complications such as hypoxia, bradycardia, and bronchopulmonary risk were present. So, the use of various types of surfactants including natural ones such as BLES, Infasurf, Curosurf, and Survanta and synthetic ones such as Surfaxin and Exosurf was amended. These surfactants are expensive, and are not available for everyone especially in countries like Iran. Based on this issue, an effective surfactant with lower cost and more availability is necessary. The first Iranian surfactant called Beraksurf was made with Survanta pharmaceutically active substance. However, the effect of this surfactant on treatment of RDS and its complications is not exactly clear.

This clinical trial study is designed to compare the effect of the Canadian BLES with the Iranian Survanta as an efficient surfactant.

**Study goals and objectives**

This study aimed to compare the therapeutic outcomes of Iranian Survanta and BLES (bovine lipid extract surfactant) on RDS treatment among preterm neonates.

**Study design**

This triple-blind randomized clinical trial study was performed on hospitalized neonates in neonatal intensive care unit (NICU) of Afzalipour hospital (Kerman, Iran) between April 1, 2020 and February 1, 2021. Diagnosis of RDS, gestational age of 28-34 weeks and weight ≥1kg were considered as inclusion criteria. Congenital anomalies such as congenital cyanotic heart diseases, digestive system anomalies and chromosome abnormalities were the exclusion criteria.

RDS diagnosis and its severity was conducted based on clinical symptoms such as tachypnea, grunting, cyanosis, and decreased respiratory sounds. Also, the lung radiologic imaging (reticulonodular and bronchogram) was considered in RDS diagnosis.

**Methodology**

Two types of surfactants were used for intervention in this study. One group received BLES 135 mg/kg (produced by Blesbiochemical, Canada) and the other group received Survanta (Beraksurf®) 100 mg/kg (produced by Tekzima, Iran). Drug administration in both groups was performed intratracheally for 10 minutes. The first dose was given immediately after hospitalization, and if it was not required at that time, it was prescribed in the hospitalization period and with deterioration of RDS (this deterioration was not justifiable with other causes).

Chest radiography was performed in both groups 6 hours after the first dose. Based on radiographic findings and neonate clinical condition, second and third dose were either prescribed or unfixed.

The number of days required for the use of invasive mechanical ventilation (ventilator) and non-invasive continuous positive airway pressure (CPAP) was recorded for all neonates.

**Safety considerations**

Some complications were evaluated. These complications are PDA (assessed by echocardiography), IVH (assessed by sonography), sepsis (using blood culture), and pneumothorax (using chest X-ray; and assessed by clinical symptoms) and pulmonary hemorrhage (using chest X-ray; and assessed by clinical symptoms). The mortalities in both groups were also recorded.

**Follow-up**

This research follow up participants in hospitalization and no need to post discharge follow up.

**Data management and statistical analysis**

Sample size was calculated at 95% confidence interval (CI) and 80% power based on a similar study (9) with standard deviation (SD) 3.7 using Stata software version 12. Sixty-four participants were assigned randomly to each group.

Data analysis was performed with Stata software version 12 (StataCorp, College Station, TX, USA), and *P* value <0.05 was considered as statistically significant.

The analysis was performed in two descriptive and inferential sections. Descriptive, quantitative and categorical data were presented as mean ± standard deviation (minimum–maximum) and frequency (percentage), respectively. Based on quantitative data of comparison between the two groups, analyses were performed using independent sample t-tests. Additionally, analyses based on categorical data between the two groups were done using Chi-square test. Also, the risk ratio (RR) was calculated for complication and mortality of RDS.

**Quality assurance**

This study was based on standard treatment protocol of RDS without any changes.

**Expected outcomes of the study**

The Beraksurf treatment in our study is a cost-effective and available surfactant; based on our results, its effect on RDS complications is similar to BLES. When compared with BLES, incidence risk of some complications such as pneumothorax and pulmonary hemorrhage in RDS patients is lower with this surfactant.

**Duration of the project**

This triple-blind randomized clinical trial study was performed from April 1, 2020 to February 1, 2021

**Project management**

Fateme Sabzevari designed the initial concept, prepared the manuscript, and helped with the first draft of the manuscript. Bahareh Bahmanbijari, Zahra Daei parizi and Zahra Jamali designed the initial concept. Mahdie Eslamian participated in manuscript preparation and data collection. Fatemeh Karami Robati participated in revising manuscript.

**Ethics**

This study was conducted in accordance to the Declaration of Helsinki. The ethics committee of Kerman University of medical sciences approved this study (Code: IR.KMU.AH.REC.1400.012).

**Informed consent forms**

The approved version of the protocol must have copies of informed consent forms (ICF), both in English and in the local language in which they are going to be administered. However, translations may be carried out after the English language ICF(s) have been approved by the ERC. If the research involves more than one group of individuals, for example healthcare users and healthcare providers, a separate specifically tailored informed consent form must be included for each group. This ensures that each group of participants will get the information they need to make an informed decision. For the same reason, each new intervention also requires a separate informed consent form.
